# Model selection to achieve reproducible associations between resting state EEG features and autism

**DOI:** 10.1038/s41598-024-76659-5

**Published:** 2024-10-25

**Authors:** William E. Carson, Samantha Major, Harshitha Akkineni, Hannah Fung, Elias Peters, Kimberly L. H. Carpenter, Geraldine Dawson, David E. Carlson

**Affiliations:** 1 Department of Biomedical Engineering, Duke University, Durham, NC 27708 USA; 2 Duke Center for Autism and Brain Development, Duke University, Durham, NC 27708 USA; 3 Department of Psychiatry and Behavioral Sciences, Duke University, Durham, NC 27708 USA; 4 Duke Institute for Brain Sciences, Duke University, Durham, NC 27708 USA; 5 Department of Civil and Environmental Engineering, Duke University, Durham, NC 27708 USA; 6 Department of Biostatistics and Bioinformatics, Duke University, Durham, NC 27708 USA; 7 Department of Electrical and Computer Engineering, Duke University, Durham, NC 27708 USA

**Keywords:** Autism, Electroencephalography, EEG, Resting state, Reproducible, Reproducibility, Autism spectrum disorders, Diagnostic markers

## Abstract

A concern in the field of autism electroencephalography (EEG) biomarker discovery is their lack of reproducibility. In the present study, we considered the problem of learning reproducible associations between multiple features of resting state (RS) neural activity and autism, using EEG data collected during a RS paradigm from 36 to 96 month-old children diagnosed with autism (*N* = 224) and neurotypical children (*N* = 69). Specifically, EEG spectral power and functional connectivity features were used as inputs to a regularized generalized linear model trained to predict diagnostic group (autism versus neurotypical). To evaluate our model, we proposed a procedure that quantified both the predictive generalization and reproducibility of learned associations produced by the model. When prioritizing both model predictive performance and reproducibility of associations, a highly reproducible profile of associations emerged. This profile revealed a distinct pattern of increased gamma power and connectivity in occipital and posterior midline regions associated with an autism diagnosis. Conversely, model selection based on predictive performance alone resulted in non-robust associations. Finally, we built a custom machine learning model that further empirically improved robustness of learned associations. Our results highlight the need for model selection criteria that maximize the scientific utility provided by reproducibility instead of predictive performance.

**Supplementary Information:**

The online version contains supplementary material available at 10.1038/s41598-024-76659-5.

## Introduction

Neural association studies (alternatively referred to as brain-wide association studies, or “BWAS”) explore the relation of various features of neural activity to a given condition, stimulus, or outcome. One of the challenges of neural association studies is identifying reproducible associations^1^. This is due to numerous factors, including small sample sizes, cohort heterogeneity, and the often-large number of features used to characterize neural activity. This is especially true if connectivity features are used, as connectivity features grow quadratically with the number of recording locations.

Association studies have been used to better understand the neural basis of autism, with several studies identifying differences in various features of neural activity. Despite established differences, the nature of such differences has varied across studies. Studies have found both excess and decreased resting state (RS) electroencephalogram (EEG) spectral power associated with autism, depending on the brain region^2–4^. Furthermore, some studies have found functional hyperconnectivity in RS EEG activity in autistic children^5,6^, while others have noted hypoconnectivity^7,8^. Others have found a combination of hyper- and hypoconnectivity associations characterize autism^4,9–11^. Reproducibility with regards to EEG features associated with autism has been investigated as well, with mixed conclusions regarding whether reproducible associations can be achieved. Holiga and colleagues^12^ found patterns of functional hyper- and hypoconnectivity associated with autism that were reproduced on separate cohorts. A multi-site study^13^ found that functional connectivity features predictive of autism demonstrated limited generalizability across sites, and marginally consistent results in larger samples. Thus, despite recent efforts, identification of a reproducible and robust profile of associations between distinct features of EEG activity and autism remains an important goal.

The goal of this work was to learn an interpretable and reproducible profile of RS neural activity associated with autism. Furthermore, we aimed to learn a profile that was *quantifiably* reproducible. Efforts to identify reproducible associations for interpretation may be hindered by model selection practices that are not conducive to reproducibility. Often, models are chosen solely to maximize predictive performance. This is an acceptable practice when the end goal is to evaluate the predictive performance of the model on a hold-out test set. However, when models are deployed in a healthcare or scientific setting (e.g., predicting diagnosis of a certain disease or condition), there are often factors other than predictive performance or model generalization that should be considered. For example, in the case of a model with linear coefficients, if one intends to interpret the learned coefficients as associations in downstream analysis or for further scientific study, then these association should be reproducible and reliable. Selecting models based purely based on predictive performance does not consider reproducibility of model parameters, nor their subsequent interpretation and generalizability. Thus, this approach may not always lead to the learning of reproducible associations.

Instead of using the usual canonical model selection approach, we propose a rethinking of model selection criteria when striving for reproducible associations. Specifically, we employed a procedure for quantifying association reproducibility and the overall robustness of our modeling pipeline and used this in conjunction with predictive performance for model selection. Using this alternative model selection procedure, we learned associations by training a regularized generalized linear model to spectral power and connectivity features derived from RS EEG data collected from a sample of children diagnosed with autism and neurotypical (NT) children. RS EEG has been used to characterize brain function due in part to its ability to capture spontaneous neural activity. Development of a RS EEG biomarker of autism could provide an objective companion to established autism behavioral assessments, potentially facilitate early identification, as well as stratification and outcome monitoring in clinical trials, and elucidate autism’s neural basis^14,15^. Using the proposed model selection criteria resulted in robust and reproducible associations in exchange for a minor loss in predictive performance, whereas the canonical model selection criteria did not find reproducible associations. We interpreted the learned profile through various visualizations to determine the spectral (i.e., low or high frequency) and spatial (i.e., regional activity, connectivity profile) features associated with autism. We also employed a machine learning (ML)-based approach developed specifically to learn generalizable and reproducible linear associations based on neural spectral features. Finally, we compared our learned associations to findings from the literature and discussed relations to hypotheses related to the neural basis of autism.

## Methods

### Participants

This work considered a sample comprised of data collected from participants over four separate studies in which similar methods of EEG collection were implemented, including use of the same laboratory, experimental stimuli, and EEG acquisition and analysis methods. The full sample included 293 participants, of which 224 (76.5%) were diagnosed with autism spectrum disorder and 69 (23.5%) were neurotypical (NT). Demographics of the participants are presented in Table 1. Parents/legal guardians provided informed consent prior to study procedures. Studies were approved by the Duke University institutional review board and all methods were performed in accordance with the relevant guidelines and regulations. Each study used similar methods for diagnostic assessment. For all studies, children with known genetic syndromes (e.g. Fragile X) and pathogenic mutations or copy number variants associated with autism, or a history of epilepsy or seizure disorder were excluded. For all studies, participant Intelligence Quotient (IQ) was assessed using standardized tests, including *Differential Abilities Scales-II* (DAS-II)^16^, *Mullen Scales for Early Learning* (MSEL)^17^, *Wechsler Preschool and Primary Scale of Intelligence* (WPPSI-IV)^18^, and *Wechsler Intelligence Scale for Children* (WISC-V)^19^, based on the participant’s age. Each of the study samples that contributed to the full sample are described next.

**Table 1:** Demographic information for full participant sample.

Study Number	Diagnostic Group	*N* Total (*N* Female)	Age Range in Months, Mean (SD)	Mean Full-Scale DQ (SD)
1	Neurotypical	40 (13)	38.0–96.0, 67.5 (15.6)	115.8 (12.5)
2		29 (14)	37.0–71.0, 50.7 (10.6)	115.7 (13.4)
Total Neurotypical Participants: *N* = 69 / 293 (23.5%)				
1	Autism	85 (22)	38.0–95.0, 68.9 (15.4)	88.9 (22.1)
3		79 (16)	38.0–96.0, 70.1 (17.9)	75.4 (21.5)
4		60 (6)	48.0–96.0, 75.2 (14.6)	92.3 (17.4)

Total autistic participants: *N* = 224 / 293 (73.5%).

*Note.* Abbreviations: DQ, Developmental Quotient; SD, Standard Deviation.

Study 1: Participants were 85 autistic and 40 NT children (90 males and 35 females), 38 to 96 months old (*M* = 67.5 months, *SD* = 15.6). Autistic participants met Diagnostic and Statistical Manual of Mental Disorders, 5th ed. (DSM-5)^20^ criteria for autism spectrum disorder based on Autism Diagnostic Interview-Revised (ADI-R)^21^ and either the Autism Diagnostic Observation Schedule, Second Edition (ADOS-2)^22^ or Brief Observation of Symptoms of Autism (BOSA)^23^, administered by research-reliable clinicians. For the NT group, participants had a Full-Scale IQ ≥ 70, and non-clinically elevated scores on the Social Responsiveness Scale Second Edition (SRS-2)^23^, Attention Deficit/Hyperactivity Disorder Rating Scale (ADHD-RS)^24^, and/or Child Behavior Checklist (CBCL)^25^, not including the CBCL anxiety subscale, for which one NT participant was included. If the participant was on stimulant medication, they were required to undergo a 24-hour washout. Exclusion criteria for both groups were (1) motor or sensory impairment that would have interfered with completion of study measures, including vision-necessitating bifocals or progressive lenses, (2) history of neonatal brain damage, (3) any known environmental circumstances likely to account for autism, and/or (4) any other factor that the investigator felt would make assessment or measurement performance invalid. Additional exclusion criteria for the NT group were known or suspected developmental, neurological, or psychiatric disorders or a sibling/first degree relative with autism or Attention Deficit/Hyperactivity Disorder (ADHD). Participants with a history of simple febrile seizures, or the absence of any seizure type for the past 12 months, were included. The autistic group was allowed one additional co-occurring diagnosis, not including ADHD, as indicated by the CBCL or Mini International Neuropsychiatric Interview (MINI)^26,27^. Mean Full Scale Developmental Quotient (FSDQ) for the Study 1 sample was 97.5 (SD = 23.3).

Study 2: Participants were 29 NT children (15 males and 14 females) 37 to 71 months old (*M* = 50.7 months, *SD* = 10.6). Participants were required to have scores on the Strengths and Difficulties Questionnaire within the normal range for all scales^28–30^. Exclusion criteria were (1) biological sibling or parent diagnosed with autism or developmental delay (DD), (2) sensory impairment affecting vision or hearing, (3) significant motor impairment (e.g., cerebral palsy), (4) chronic or acute medical illness, and/or (5) current use of psychoactive and/or seizure medications. Mean FSDQ for the Study 2 sample was 115.7 (*SD* = 13.4).

Study 3: Participants were 79 autistic children (63 males and 16 females), 38 to 96 months old (*M* = 70.0 months, *SD* = 17.9). Data were collected at the baseline visit of a clinical trial evaluating efficacy of umbilical cord blood for improving social communication skills in autistic children (ClinicalTrials.gov: NCT02847182). Participants were required to be aged 24 to 97 months and met DSM-5 criteria for autism spectrum disorder based on ADOS-2 and ADI-R, administered by research-reliable clinicians. Inclusion criteria were (1) stability on current medications for at least 2 months, (2) participants and parents/guardians were English-speaking, and (3) an autologous umbilical cord blood unit or >/= ¾ HLA-matched allogeneic, unrelated umbilical cord blood unit was available. Exclusion criteria were (1) history of prior cell therapy, (2) use of intravenous immunoglobulin or other anti-inflammatory medications (with the exception of NSAIDs), (3) a significant psychiatric co-occurring conditions, (4) any of the following medical conditions: metabolic disorders, mitochondrial dysfunction, any seizure disorder, cancer, chemotherapy, immunodeficiency disorders, autoimmune cytopenias, significant sensory or motor impairment, coexisting medical conditions that put child at risk for sedation, hematologic abnormalities, genetic or acquired diseases or comorbidities that could have required future stem cell treatment, (5) clinically relevant physical dysmorphology indicative of genetic syndrome as assessed by the principal investigators, (6) significantly impaired renal or liver function, (7) known active infection, (8) sibling enrolled in the same study, and/or (9) clinically significant abnormalities in complete blood count. Mean FSDQ for the Study 3 sample was 75.4 (*SD* = 21.5).

Study 4: Participants were 60 autistic children (54 males and 6 females), 48 to 96 months old (*M* = 75.2 months, *SD* = 14.6). Data were collected at the baseline visit of a clinical trial evaluating efficacy of mesenchymal stromal cells in improving social communication skills for autistic children (ClinicalTrials.gov: NCT04089579). Participants met DSM-5 criteria for autism spectrum disorder based on ADI-R and either ADOS-2 or BOSA, administered by research-reliable clinicians, and had a Full-Scale IQ or General Abilities Index score ≥ 65. Inclusion criteria were (1) stability on current medications for at least 2 months, (2) normal lymphocyte count, and (3) participant and family were English-speaking. Exclusion criteria were (1) known diagnoses of any of the following coexisting psychiatric conditions: depression, bipolar disorder, schizophrenia, obsessive-compulsive disorder associated with bipolar disorder, or Tourette syndrome, (2) sibling enrolled in the same study, (3) active or uncontrolled infection, (4) recent exposure to COVID-19, (5) any of the following medical conditions: metabolic disorders, mitochondrial dysfunction, any seizure disorder, cancer, immunodeficiency disorders, autoimmune cytopenias, significant sensory or motor impairment, impaired renal or liver function, hematologic abnormalities, or genetic or acquired diseases or comorbidities that could have required future stem cell treatment, (6) clinically relevant physical dysmorphology indicative of genetic syndrome as assessed by principal investigators, (7) coexisting medical conditions that placed child at increased risk for complications of study procedures, (8) prior history of cell therapy, (9) current or prior use of IVIG or other anti-inflammatory medications (excluding NSAIDs), (10) current or prior immunosuppressive therapy, and (11) participant had their own supply of banked cord blood or parents declined the use of qualified, donor-matched cord blood unit. Mean FSDQ for the Study 4 sample was 92.3 (*SD* = 17.4).

### EEG data collection and processing

High-density, continuous EEG data were collected at a sampling rate of 1000 Hz while children watched three videos: (1) a silent video depicting floating bubbles; (2) a woman reciting nursery rhymes, and (3) brightly colored sound-making dynamic toys. Each video lasted 60 to 65 seconds. Video viewing order was counterbalanced across participants to eliminate potential order effects. For analysis of RS EEG discussed in this work, only recordings taken during participant viewing of the floating bubbles video were included in the analyses. This neutral type of visual stimulus with no accompanying sound has been previously used to elicit a “resting state-like” condition for EEG data collection in young children^31,32^. EEG data were recorded from 124 electrodes using a Hydrocel Geodesic Sensor Net (EGI) and Net Amps 400 amplifier in Net Station 4.5.6 or 4.5.7.

EEG data were processed using the Harvard Automated Processing Pipeline for Electroencephalography (HAPPE) version 3.2^33^ in MATLAB 2019b. EEG artifacts were removed via a multi-step process. The steps used by HAPPE to process EEG data were as follows: (1) three outer-ring electrode channels were excluded leaving 121 electrodes, (2) line noise and harmonics were reduced (60, 120, 180, 240, and 300 Hz) using CleanLine^34^, (3) bad channels were detected and interpolated, (4) wavelet thresholding was employed, (5) data were segmented to 1-second epochs, (6) segment rejection was performed using both amplitude (-100 to 100 *u*V) and segment similarity, and (7) data were re-referenced to an average reference over 121 electrodes. Processed EEG was partitioned into 1-second-long epochs. Epochs corresponding to moments of inattention were discarded. Participants were included in the subsequent analyses if at least 1/3 of original epochs were retained and less than 1/3 of electrode channels were interpolated during HAPPE processing. A 19-channel subset of electrodes corresponding to the international 10–20 system of EEG recording^35^ were selected for inclusion in subsequent analysis. This subset was chosen to align with recording layouts used in recent large-scale EEG studies of children with autism^14^.

### Power and cross-power feature generation

Power spectral density and cross-power spectral density features (subsequently referred to as “power” and “cross-power,” for brevity) were generated using a multitaper estimation method^36^ from 1 to 55 Hz in 1-Hz-increments for each 1-second-long window of neural activity. Multitaper spectral estimation uses multiple orthogonal Slepian basis vectors as tapers to compute. Multitaper cross-power between channels $\:i$ and $\:j$ (denoted as $\:{\widehat{S}}^{ij}\left(f\right)$) is computed as the average of $\:K$ separate cross-spectral estimates:

1 $$\:{\widehat{S}}^{ij}\left(f\right)=\frac{1}{K}\sum\limits_{k=1}^{K}{\widehat{S}}_{k}^{ij},$$

where $\:f$ represents a given frequency. The equation for the $\:k$-th multitaper spectral estimate $\:{\widehat{S}}_{k}^{ij}$ is as follows:

2 $$\:{\widehat{S}}_{k}^{ij}\left(f\right)={\left|\sum\limits_{t=1}^{T}{\varvec{s}}_{k}\left[t\right]\varvec{x}\left[t\right]{e}^{-j2\pi\:ft}\right|}^{2},$$

where $\:{\varvec{s}}_{k}\left[t\right]$ represents an $\:T$-element Slepian basis vector evaluated at the $\:t$-th sample, and $\:\varvec{x}\left[t\right]$ represents a $\:T$-element time series signal evaluated at element $\:t$^37^. Power spectral density and cross-power spectral density features were generated using a multitaper estimation method using functions provided by the MNE library^38^ in Python. For this analysis, five orthogonal tapers were used to generate multitaper features. Cross-power estimation over signal collected from $\:L$ separate regions produced an $\:L\times\:L$ non-negative, symmetric connectivity matrix detailing the functional connectivity for each frequency f. To eliminate redundant features, we retained features from the upper triangular of cross-power features plus the diagonal of power features for a given frequency. Using power and cross-power in-tandem facilitates characterization of region-specific activity and functional connectivity between regions in terms of features with similar properties, distributions, scales, and variation. This resulted in a total of 10,450 features generated per neural time series window (190 features per frequency, 55 frequencies). Data augmentation was performed to provide our model with additional exposure to variance in the neural data. For each 1-second-long window, multiple spectral cross-power estimates were made using shorter, partially overlapping windows within the 1-second-long window. To approximately balance classes, more overlapping windows were used in augmentation of NT samples. Data augmentation produced 134,155 NT participant samples and 130,760 samples from participants diagnosed with autism for a total of 264,915 samples.

### Logistic regression classification

Using power and cross-power features as input, a logistic regression model with an L2-regularization penalty was trained in a binary classification task to predict a diagnosis of autism vs. NT. The objective of L2-regularized logistic regression is as follows:

3 $$\mathcal{L} =\min\limits_{w,b}\;\sigma\:\left(w^{\mathrm\tau}\;\log\;x+b\right)+\lambda\:\left\|w\right\|^2,$$

where $\:\varvec{w}\in\:{\mathbb{R}}^{p}$ represents the $\:p$-dimensional linear coefficients (i.e. associations), $\:b\in\:\mathbb{R}$ represents the bias, $\:\lambda\:$ represents a positive hyperparameter used to weight the relative importance of the regularization penalty in the objective, and $\:\sigma\:\left(\cdot\right)$ represents the sigmoid activation function, $\:\sigma\:\left(a\right)=1/(1+\text{e}\text{x}\text{p}\left(-a\right))$. Here, we are interested in interpreting the associations detailed by the coefficients $\:\varvec{w}$. $\:\varvec{x}\in\:{\mathbb{R}}_{+}^{p}$ represents non-negative spectral features derived from the RS EEG data. Although it is possible to learn associations with respects to non-log-transformed features, we found empirically that log-transforming features resulted in much higher reproducibility and stability of learned associations. Additionally, since associations were found with respects to a high-dimensional feature space, we used L2 regularization to limit the expressivity of the logistic regression classifier. An otherwise unregularized classifier may be able to overfit with respect to this high feature space, resulting in poor generalization.

All L2-regularized logistic regression models were trained in Python (v3.9.16) using implementations provided by the scikit-learn (v1.2.2) machine learning library. A search was performed over the regularization strength hyperparameter (denoted as $\:C$ in the scikit-learn L2-regularized logistic regression implementation, with values of $\:C$ being inversely proportional to regularization strength) for L2-regularized logistic regression models. The search was executed by incrementally modifying the regularization strength from a value of $\:1\cdot\:10^{-5}$ to $\:1\cdot\:10^{-12}$ in increments of $\:{10}^{-1}$ in $\:\text{log}$-space for a total of eight regularization strength values tested (smaller $\:C$ values correspond to greater regularization). A stopping criteria tolerance (“tol” hyperparameter in the scikit-learn logistic regression implementation) value of $\:1\cdot\:10^{-3}$ was used. A maximum number of solver iterations (denoted as “max_iter” in the scikit-learn logistic regression implementation) of $\:1\cdot10^{-2}$ was used. The default values were used for all other logistic regression hyperparameters.

### Evaluating and selecting for model robustness

**Figure 1:**
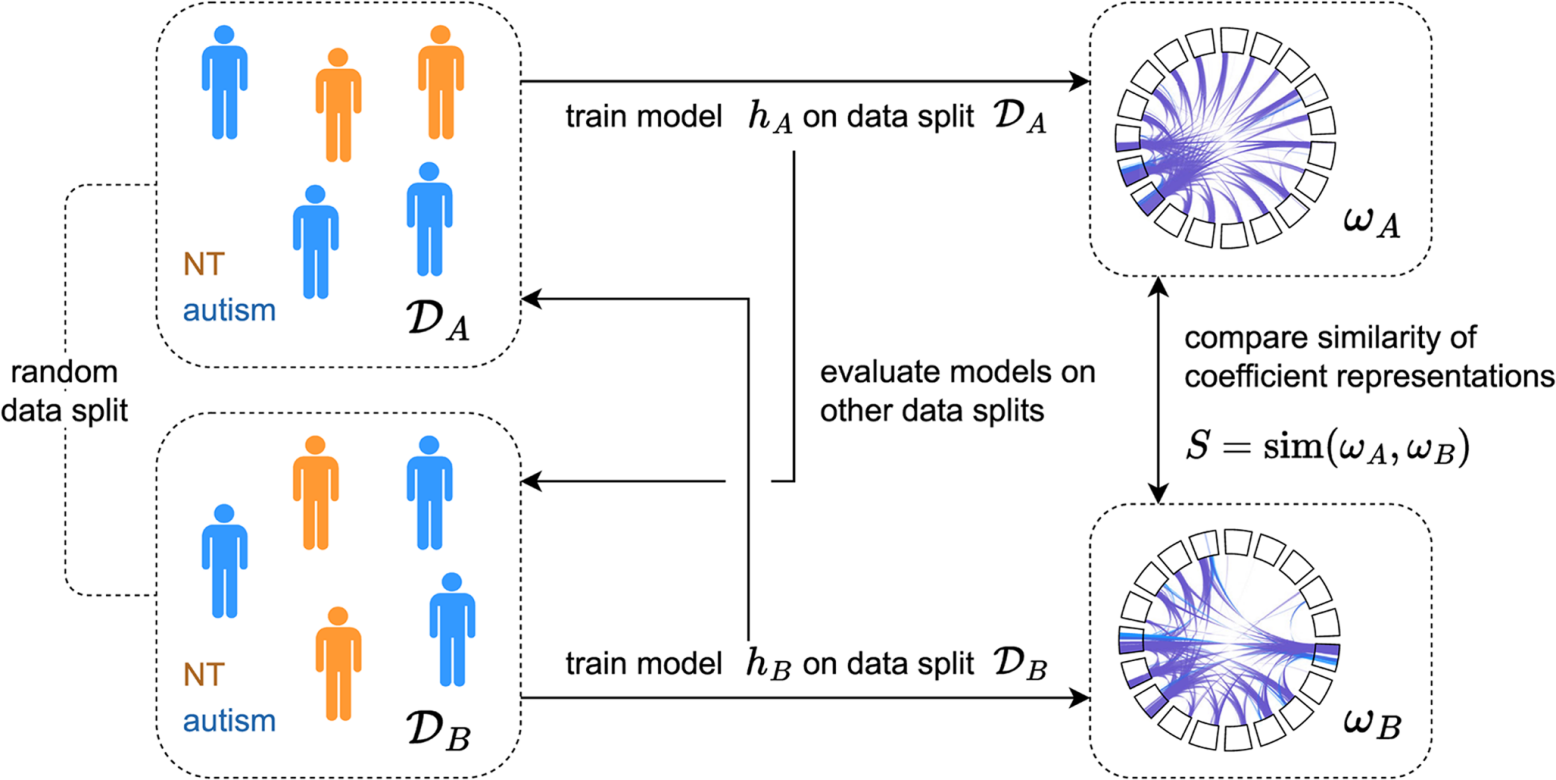
Diagram of model robustness evaluation procedure. Given a dataset $\:\mathcal{D}$, predictive performance metric $\:P\in\:\left[-\text{1,1}\right]$, similarity measure $\:S\in\:\left[-\text{1,1}\right]$, and a model with linear coefficients $\:\varvec{w}$, data is first partitioned into two disjoint sets, $\:{\mathcal{D}}_{A}$ and $\:{\mathcal{D}}_{B}$. The model is trained twice – once on each set – and tested on the set on which it was not trained. The two test performances ($\:{P}_{A}$ and $\:{P}_{B}$) are averaged to get the predictive performance $\:P=\left({P}_{A}+{P}_{B}\right)/2$ of the model. The similarity of the coefficient representations is then quantified using the chosen similarity metric, $\:S=\text{s}\text{i}\text{m}\left({\varvec{w}}_{A},{\varvec{w}}_{B}\right)$. Model robustness $\:R$ is calculated as the multiplicative product of predictive performance $\:P$ and representational similarity $\:S$: $\:R=P\cdot\:S$.

We evaluated our modeling pipeline according to a procedure meant to quantify “robustness” of a model with linear coefficients. The procedure is as follows: given a dataset $\:\mathcal{D}$, a predictive performance metric $\:P\in\:\left[\text{0,1}\right]$ (e.g. AUROC), a similarity measure $\:S\in\:\left[-\text{1,1}\right]$ (e.g. Spearman’s rank correlation coefficient), and a model with linear coefficients $\:\varvec{w}$, data is first partitioned into two disjoint sets, $\:{\mathcal{D}}_{A}\subseteq\:\mathcal{D},{\mathcal{D}}_{B}\subseteq\:\mathcal{D},{\mathcal{D}}_{A}\cap\:{\mathcal{D}}_{B}=\varnothing\:$. The model is trained twice – once on each set – and tested on the set on which it was not trained. The two test performances ($\:{P}_{A}$ and $\:{P}_{B}$) are averaged to get the predictive performance $\:P$ of the model. The similarity of the coefficient representations is then quantified using the chosen similarity metric, $\:S=\text{s}\text{i}\text{m}\left({\varvec{w}}_{A},{\varvec{w}}_{B}\right)$. If the representations are similar, the model is said to exhibit high “representational reproducibility,” as the model learned similar coefficient representations on two different, non-overlapping sets of data (e.g., data collected from two different studies). Without both adequate performance and representational reproducibility, we cannot be confident in interpreting our learned linear representation. Thus, we posit that model robustness $\:R$ should be the multiplicative product of predictive performance $\:P$ and representational similarity $\:S$:

4 $$\:R=\frac1C{\sum\:}_{c=1}^CP_c\cdot\:S_c,$$

where $\:C$ represents the number of repetitions over which model robustness was evaluated, and $\:{P}_{c}$ and $\:{S}_{c}$ represent model predictive performance and representational similarity achieved on disjoint set $\:c$. Alternatively, this procedure can be thought of as $\:C$ repetitions of a 2-fold CV evaluation procedure. For this work, we used $\:C$ = 10 repetitions of disjoint data splits to evaluate model robustness, with AUROC used as the predictive performance metric and Spearman’s rank correlation coefficient as the metric for representational similarity. We rescale AUROC as $\:P\equiv\left(2\cdot AUROC\right)-1$ so that a model with poor performance corresponds to a near-zero value. Model robustness was evaluated for a range of regularization values for L2-regularized logistic regression. In addition to AUROC, we reported class-balanced accuracy, sensitivity, and specificity. Performance metrics indicate performance for prediction of participant diagnosis (not window-wise prediction). A diagram depicting our procedure for quantifying model robustness is provided in Fig. 1. For more details on the procedure for quantifying model robustness, see Algorithm 1. Notably, this procedure can be generalized based on the goals and structure of the study. For example, a different model with linear parameters or coefficients can be used/evaluated, and different metrics used to quantify predictive performance and reproducibility.

**Figure:**
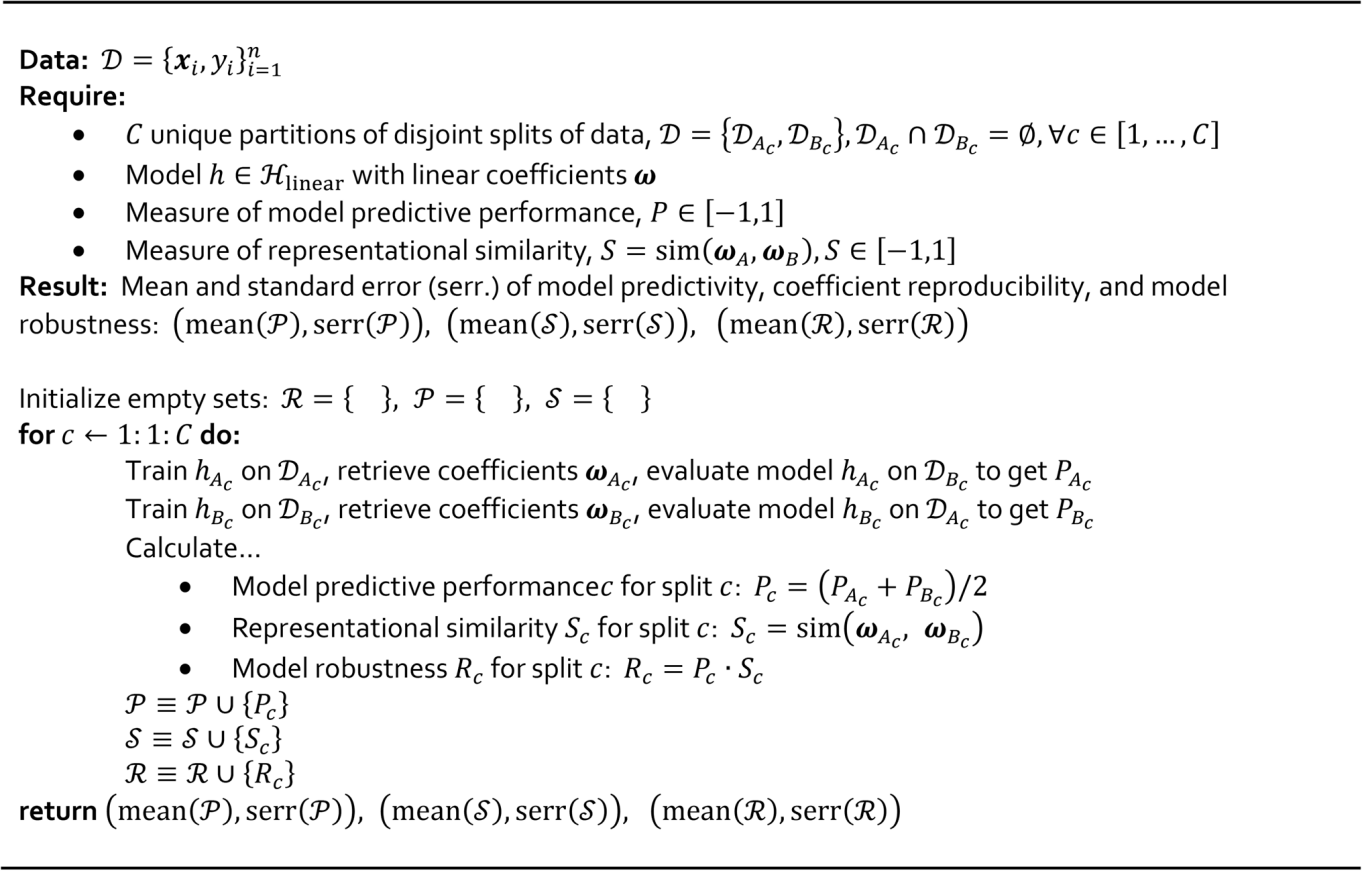
Algorithm 1. Algorithm for quantifying robustness of a model with linear coefficients. The algorithm also quantifies model predictivity and reproducibility of the linear coefficients.

### Experimental design and statistical analyses

We predicted either a diagnosis of autism ($\:y=1$) or NT ($\:y=0$) using features derived from a given window of neural activity. Models were trained and evaluated according to splits *over subjects*. That is, observations for a given participant were all assigned either to the training set or the test. This simulates a real-life application in which a model would be trained on data collected from a cohort of individuals and later used to make predictions for new, unobserved individuals. Prediction of participant diagnosis was calculated as the mean of the predictions made for all windows of activity from a given participant (as there were multiple windows of neural activity per participant). Reported predictive performance and coefficient reproducibility results correspond to performances achieved with model parameters resulting in the highest model robustness.

We performed an analysis to determine whether our model captured any relationships beyond participant diagnosis. For a randomly chosen repetition within the model robustness evaluation procedure, we calculated the Pearson correlation coefficient of model prediction logits aggregated over test folds with participant clinical variables, including participant age and FSDQ. A Bonferroni correction was applied to account for multiple comparisons. Correlation was determined to be significant if *p*-values were below a significance threshold of *α* = 0.05.

### Machine learning feature preprocessing

In addition to evaluating logistic regression performance, we evaluate a ML approach designed to increase reproducibility of associations. Our modeling pipeline includes (1) a feature preprocessing step intended to correct for sources of variance unrelated to our signal of interest (i.e., signal associated with autistic brain activity) and (2) input of these preprocessed features into an L2-regularized logistic regression classifier.

We refer to the feature preprocessing method used in this work as SIRT: Scale-Invariant Residuals Transform. SIRT is based on concepts from the signal processing literature. SIRT transforms non-negative input features (denoted $\:\varvec{x}$) to new space (denoted $\:\stackrel{\sim}{x}$) by leveraging relationships specific to the modeling of power spectra. This transformation accomplishes two things. First, it places features on approximately the same scale. Second, the transformation partially corrects for sources of variation that may obscure variance related to the signal of interest. In doing so, SIRT preprocessing facilitates learning more robust and reproducible linear coefficient representations. Additionally, SIRT maintains the non-negativity of features, $\:\stackrel{\sim}{\varvec{x}}>0$. SIRT is composed of a model that approximates the data from a lower-dimensional space (i.e. an autoencoder). To model the data, SIRT employs an autoencoder comprised of an encoder and decoder. The objective of training the SIRT autoencoder is expressed as follows:

5 $$\:{\mathcal{L}}_{\text{t}\text{o}\text{t}\text{a}\text{l}}={D}_{\text{I}\text{S}}\left(\varvec{x}\parallel\:\widehat{\varvec{x}}\right).$$

$\:{D}_{\text{I}\text{S}}\left(\varvec{x}\parallel\:\widehat{\varvec{x}}\right)$ represents the Itakura-Saito divergence (IS divergence)^39^ of the data reconstruction $\:\widehat{\varvec{x}}$ from the input data $\:\varvec{x}$. The IS divergence is given as follows:

6 $$\:{D}_{\text{I}\text{S}}\left(\varvec{x}\parallel\:\widehat{\varvec{x}}\right)=\frac{\varvec{x}}{\widehat{\varvec{x}}}-\text{ln}\frac{\varvec{x}}{\widehat{\varvec{x}}}-1.$$

IS divergence has the desirable property of being scale invariant. This means that the same relative weight is given to reconstruction of features of varying scale. This scale-invariant property is relevant to neuroscience applications as the magnitude of many measures used to characterize neural activity (e.g., spectral power, cross-spectral power) decay at a rate approximately inversely related to increasing frequency $\:f$, or approximately $\:1/f$. If feature scaling is not performed prior to analysis, a non-scale invariant distance metric (e.g., mean squared error) would prioritize reconstruction of low- frequency features, omitting higher-frequency information from the learned representations. Differences in feature scale can be addressed by normalizing features prior to training. However, we note that IS divergence implements a noise model better suited for non-negative data, especially spectral features^39,40^. When IS divergence is used to quantify the distance of the reconstructed data $\:\widehat{\varvec{x}}$ from the data $\:\varvec{x}$, the residuals $\:{\varvec{\epsilon\:}}_{\varvec{x}}\in\:{\mathbb{R}}_{+}^{m}$ are multiplicative with respect to the data and gamma-distributed:

7 $$\:\varvec{x}=\widehat{\varvec{x}}\circ\:{\varvec{\epsilon\:}}_{\varvec{x}},$$

where $\:\circ\:$ represents element-wise multiplication. By rearranging Eq. 7, we can define the empirical reconstruction residuals $\:{\varvec{\epsilon\:}}_{\varvec{x}}$ as follows:

8 $$\:{\varvec{\epsilon\:}}_{\varvec{x}}=\frac{\varvec{x}}{\widehat{\varvec{x}}}.$$

Following training of the autoencoding model in which the autoencoder produces a low-dimensional approximation of the data $\:\widehat{\varvec{x}}$, the SIRT transform is given as follows:

9 $$\:\stackrel{\sim}{\varvec{x}}=\frac{\varvec{x}}{\widehat{\varvec{x}}}.$$

Comparison of Eqs. 8 and 9 reveal that the empirical reconstruction residuals $\:{\varvec{\epsilon\:}}_{\varvec{x}}$ and the definition of SIRT-transformed features $\:\stackrel{\sim}{\varvec{x}}$ are in fact equivalent. Thus, training a model on SIRT-transformed features $\:\stackrel{\sim}{\varvec{x}}$ is equivalent to training on the empirical reconstruction residuals $\:{\varvec{\epsilon\:}}_{\varvec{x}}$ produced by an autoencoding model whose data reconstruction objective is the IS divergence. Properties of $\:\stackrel{\sim}{\varvec{x}}$ depend on the “capacity” of the SIRT autoencoder model and how much variance is explained by the model. If the capacity of the autoencoding model is large (i.e., a large hidden dimension or “bottle neck”), then little of the original variance in the data $\:\varvec{x}$ will be expressed in the residuals $\:\stackrel{\sim}{\varvec{x}}$. Conversely, if the capacity of the autoencoding model is small (i.e., a small hidden dimension or “bottle neck”), then a majority of the original variance in the data $\:\varvec{x}$ will be expressed in the residuals $\:\stackrel{\sim}{\varvec{x}}$. In summary, $\:\stackrel{\sim}{\varvec{x}}\in\:{\mathbb{R}}_{+}^{m}$ represents non-negative, transformed versions of the original non-negative features $\:\varvec{x}\in\:{\mathbb{R}}_{+}^{m}$. Instead of regressing directly on $\:\varvec{x}$, the logistic regression term finds a coefficient representation with respect to the log-transform of the SIRT preprocessed features $\:\stackrel{\sim}{\varvec{x}}$.

**Figure 2:**
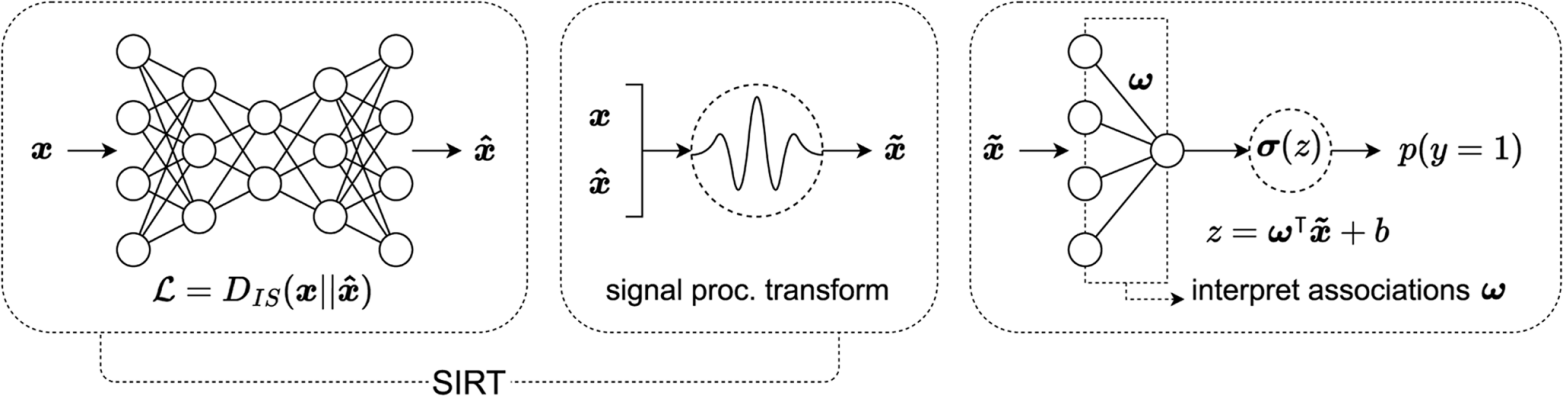
ML modeling pipeline diagram. First, an autoencoder is trained to approximate the data $\:\varvec{x}$ from a low-dimensional latent space using the Itakura-Saito divergence (IS divergence) as the reconstruction objective. After training the autoencoder, both the data $\:\varvec{x}$ and data reconstruction $\:\widehat{\varvec{x}}$ are used in a deterministic transform based on signal processing relationships to produce a non-negative, transformed version of the data $\:\stackrel{\sim}{\varvec{x}}$. A logistic regression classifier is then trained to predict either diagnosis of autism or neurotypical (NT) using the transformed version of the data $\:\stackrel{\sim}{\varvec{x}}$ as input. Finally, after logistic regression classifier training the logistic regression coefficients $\:\varvec{\omega\:}$ are taken and used to interpret the learned associations with autism diagnosis or NT diagnosis.

In this work, we used number of components/latent dimension of size 15 for the SIRT autoencoding model. A small number of components was chosen for multiple reasons. For one, we wanted the SIRT-transformed features $\:\stackrel{\sim}{\varvec{x}}$ to maintain a majority of variance of the original data $\:\varvec{x}$. However, we wanted to also account for principal sources of variance that we hypothesized were unrelated to our signal of interest (i.e., signal associated with diagnosis of autism). We hypothesized that the first few principal components primarily represented individual-specific variation (e.g., different participant physiological characteristics, participant age, etc.) that were unrelated to signal associated with diagnosis of autism. Thus, the SIRT feature transform in Eq. 7 is equivalent to removal/mitigation of the principal sources of spectral variation captured by the autoencoding model trained to minimize an IS divergence objective. A diagram depicting the modeling pipeline procedure used in this work is provided in Fig. 2.

Critically, in our model robustness evaluation procedure a new SIRT autoencoder model was trained for each set of data every time a data split was performed and evaluated on the set on which it was not trained. That is, neither the SIRT autoencoder model nor the logistic regression classifier had any access to data in from test set prior to test set evaluation.

We employed a SIRT autoencoding model with a multi-layer, fully connected neural network-parameterized encoder, with 3 hidden layers of dimensions 128, 64, 32, respectively. Each hidden layer of the neural network encoder was followed by a batch normalization layer^41^ and a dropout^42^ layer with a dropout probability of 0.7. For the decoder, non-negative matrix factorization (NMF) was used with a 15-component latent dimension. Both latent encodings as well as component elements were constrained to be non-negative. All SIRT models were implemented and trained in Python (v3.9.16) using the PyTorch (v2.0.0) deep learning framework. SIRT autoencoder parameters were optimized using a variation of batch stochastic gradient descent, specifically, the Adam optimizer^43^. Adam with weight decay (referred to as “AdamW” in PyTorch) was used as the optimizer with the default hyperparameters provided by PyTorch. The parameters of the neural network encoders were subjected to a weight decay value of $\:{10}^{-1}$. For model robustness evaluation, the SIRT autoencoder was trained for 10 epochs. For a given training fold, the model parameterization that achieved the lowest per-epoch training loss was checkpointed and later loaded and evaluated on the test set. Observations were batched according to an age inverse propensity-weighted sampling procedure to account for age imbalances in our cohort. This prevented the model from using spurious correlations of diagnosis with age of participants for prediction of diagnosis.

## Results

### Model performance, robustness, and coefficients interpretations

**Figure 3:**
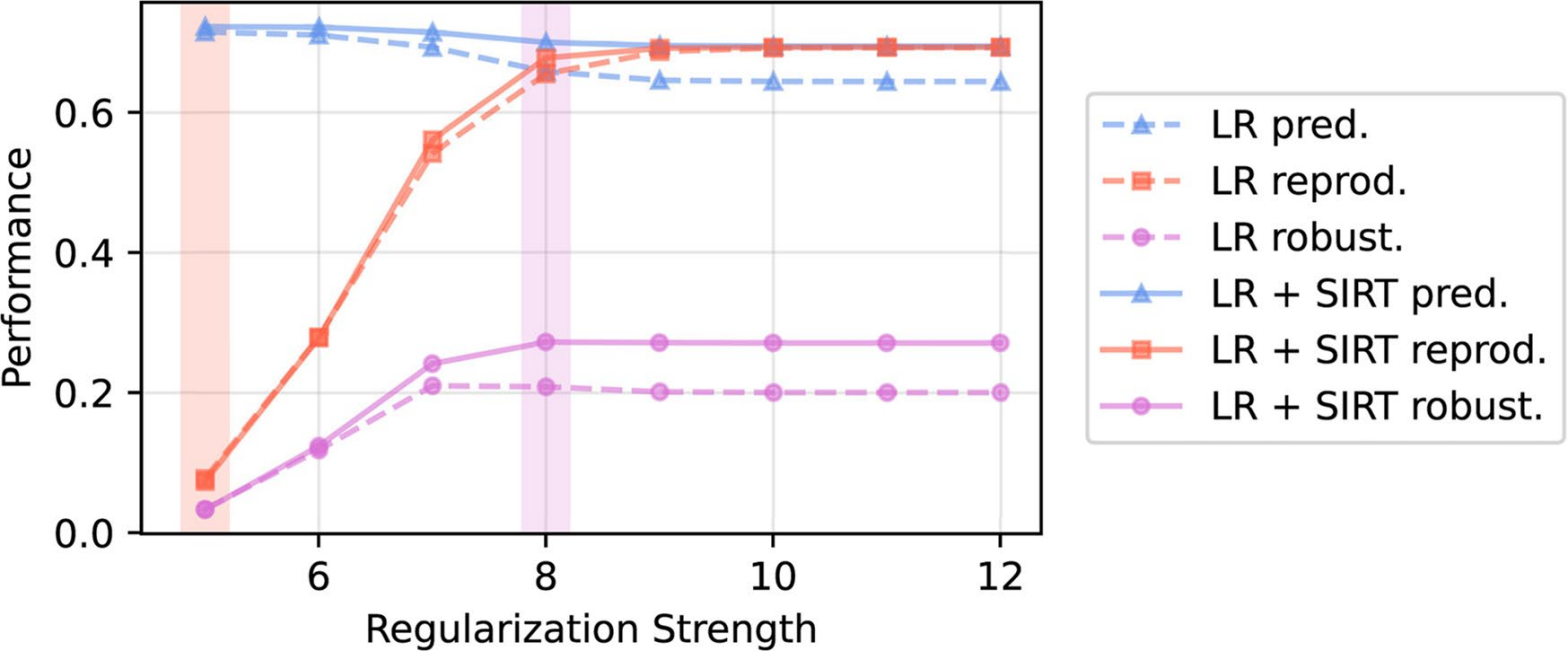
Model predictivity (quantified by area under the receiver operating characteristic, or “AUROC”), coefficient reproducibility (quantified by Spearman’s rank correlation coefficient), and overall model robustness as a function of L2-regularized logistic regression regularization strength. The model (logistic regression, or “LR,” + scale invariant residuals transform, or “SIRT,” preprocessing) corresponding to the highest achieved robustness score is highlighted by the vertical magenta band. The model corresponding to the highest achieved predictivity is highlighted by the vertical red band. $\:x$-axis values represent increasing L2 regularization strength (from left to right), corresponding to the negative $\:{\text{log}}_{10}$-transformed regularization hyperparameter values used in scikit-learn’s implementation of L2-regularized logistic regression.

L2-regularized logistic regression was trained to predict a diagnosis of autism vs. NT. When logistic regression models were selected according to predictive performance alone, the model achieved an average AUROC of 0.72 ± 0.01, a representational reproducibility score of 0.07 ± 0.01, and an overall model robustness score of 0.03 ± 0.01. When models were selected according to overall robustness, the model achieved an AUROC of 0.69 ± 0.01, a representational reproducibility score of 0.54 ± 0.02, and an overall model robustness score of 0.21 ± 0.01. Thus, selecting for model robustness led to an increase in representational reproducibility of over 600% and an over 500% increase in model robustness compared to that achieved when selecting for model predictivity alone. A visualization of model predictivity, representational reproducibility, and model robustness as a function of regularization strength is provided in Fig. 3. In general, regularization strength greatly improved reproducibility of learned associations as well as model robustness.

When trained to predict a diagnosis of autism vs. NT, L2-regularized logistic regression on SIRT preprocessed features achieved an AUROC of 0.70 ± 0.01. Coefficients learned on SIRT-preprocessed features achieved a representational reproducibility score of 0.68 ± 0.02, a 25% improvement in representational reproducibility over that learned on non-SIRT preprocessed features when selecting models for overall robustness. Logistic regression paired with SIRT preprocessing achieved a model robustness score of 0.27 ± 0.01, approximately 30% improved over logistic regression alone (model robustness 0.21 ± 0.01). Thus, our proposed ML approach led to an empirical increase in both representational reproducibility and model robustness. Diagnostic predictive performance, model robustness, and representational reproducibility results are reported in Table 2.

**Table 2:** Model predictive performance, representational reproducibility, and model robustness when trained to predict participant diagnosis of autism vs. NT, evaluated according to the procedure for determining model robustness. 10 repetitions of disjoint data splits were used to quantify model robustness metrics. Results achieved with model parameters corresponding to the highest model robustness score for a given modeling pipeline. Standard error (SE) reported.

Model	Predictive Performance (SE)	Representational Reproducibility (SE)	Model Robustness (SE)
Logistic Regression	0.693 ± 0.004	0.542 ± 0.019	0.210 ± 0.010
Logistic Regression + SIRT	0.700 ± 0.004	0.678 ± 0.016	0.272 ± 0.010

*Note.* Abbreviations: SIRT, Scale-Invariant Residuals Transform; AUROC, area under the receiver operating characteristic curve; SE, standard error.

**Figure 4:**
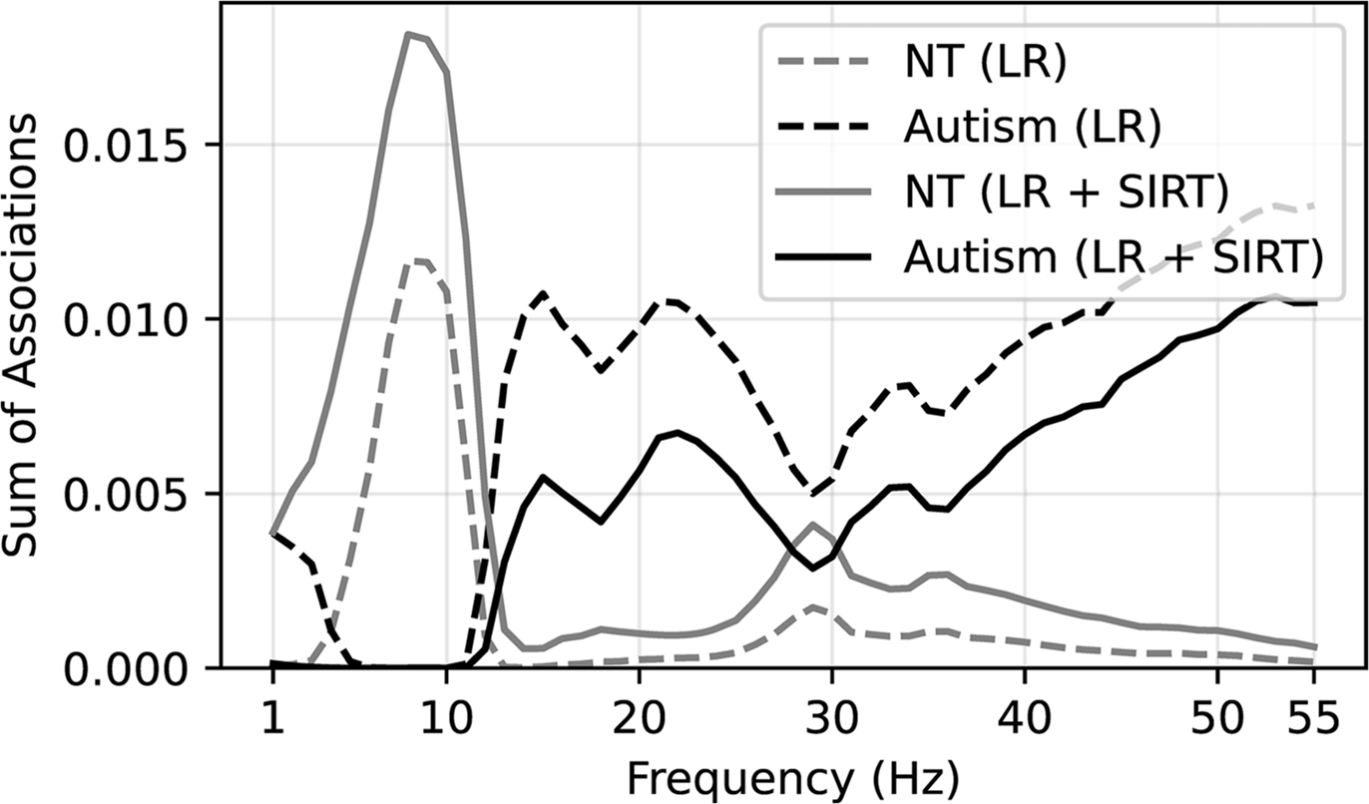
Sum of coefficient associations as a function of frequency. Coefficient values greater than zero are associated with autism diagnosis, and coefficient values less than zero are associated with NT classification. Absolute value of negative coefficient values associated with NT classification is taken for sake of comparison. Coefficient values associations are summed for a given frequency.

**Figure 5:**
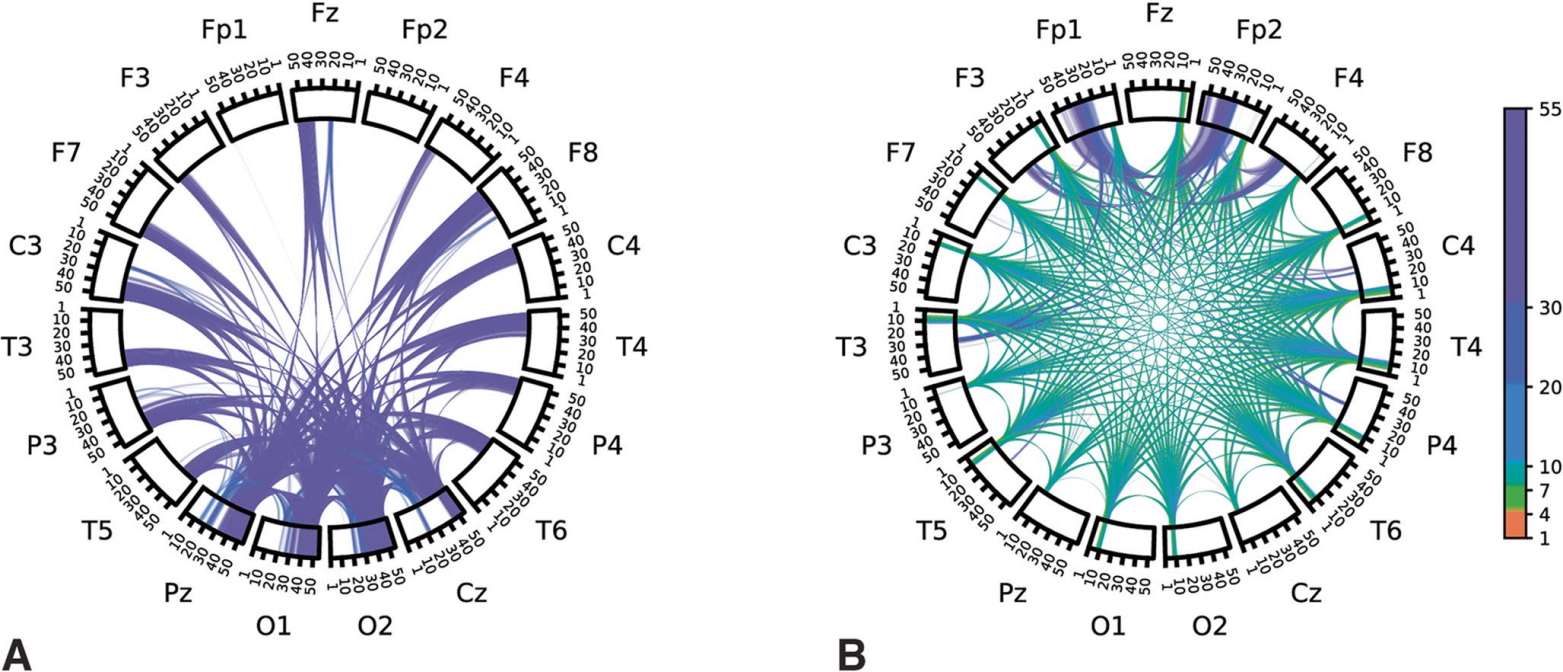
(**a**) Chord plot of learned spectral power and cross-power features associated with children diagnosed with autism (i.e., top *positive* coefficient values) learned by the model (logistic regression + SIRT preprocessing) with the highest model robustness score. (**b**) Chord plot of features associated with neurotypical (NT) children (i.e., top *negative* coefficient values) learned by the model with the highest model robustness score. Labels along the outer perimeter indicate electrodes from which neural activity was recorded. Colored bands within rectangular segments directly below electrode labels depict regional power at different frequencies associated with autism. Cross-power features associated with autism is represented as bands/arcs traversing the plot from one recording region to another. Different colors are used to indicate canonical frequency bands to aid in discrimination of activity at different frequencies. A threshold was applied in which the top 5% percent of coefficient values.

**Figure 6:**
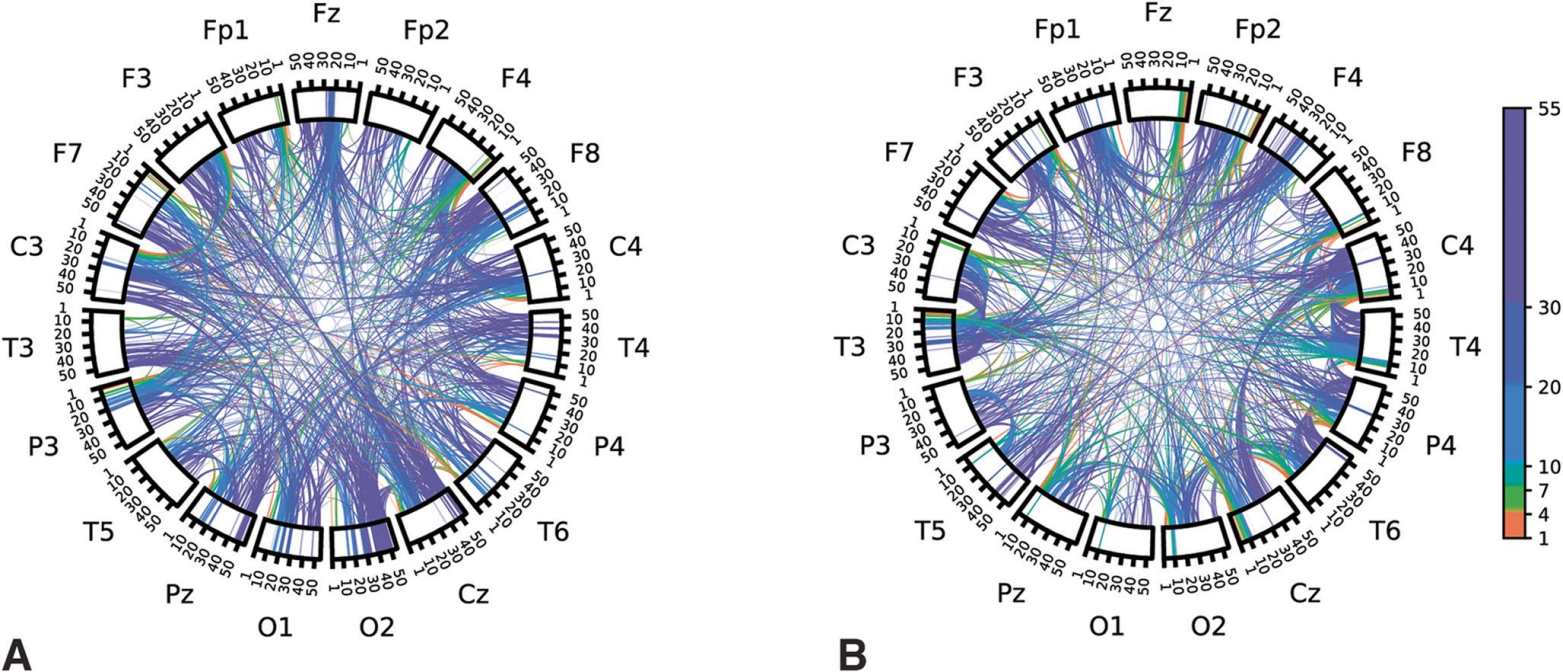
(**a**) Chord plot of learned spectral power and cross-power features associated with children diagnosed with autism (i.e., top *positive* coefficient values) learned by the model (logistic regression + SIRT preprocessing) with the highest predictive performance. (**b**) Chord plot of features associated with neurotypical (NT) children (i.e., top *negative* coefficient values) learned by the model with the highest predictive performance. A threshold was applied in which the top 5% percent of coefficient values were visualized to aid in visual clarity.

**Figure 7:**
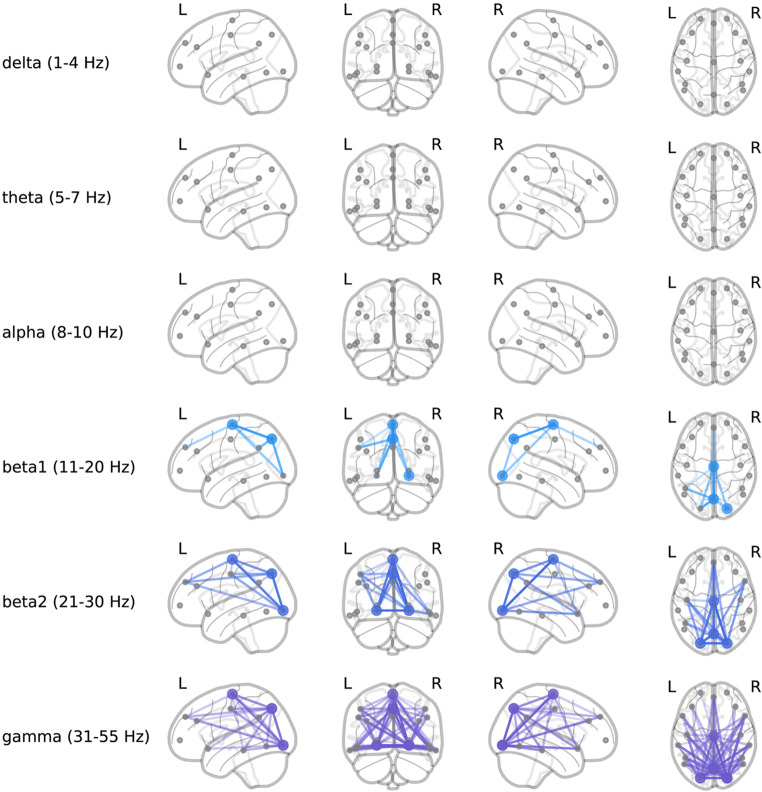
Brain view schematics of learned spectral power and cross-power features associated with autism (Fig. 5a companion). Associations are averaged over each frequency band and plotted on left sagittal (first column), coronal (second column), right sagittal (third column), and axial (fourth column) views of the brain. Each row represents associations of a different frequency band. Gray dots represent 2D projections of 3D electrode coordinate locations of 10–20 subset electrodes (e.g., gray dots in the axial view indicate Cartesian electrode coordinates). Colored, circular markers plotted over electrode locations denote power associated with autism, and colored lines spanning between electrode locations denote cross-power associated with autism. Features within the top 5% of associations were plotted for clarity. We average associations over canonical EEG bands to aid in visualization only. We note that in our approach we do not aggregate spectral features according frequency bands.

**Figure 8:**
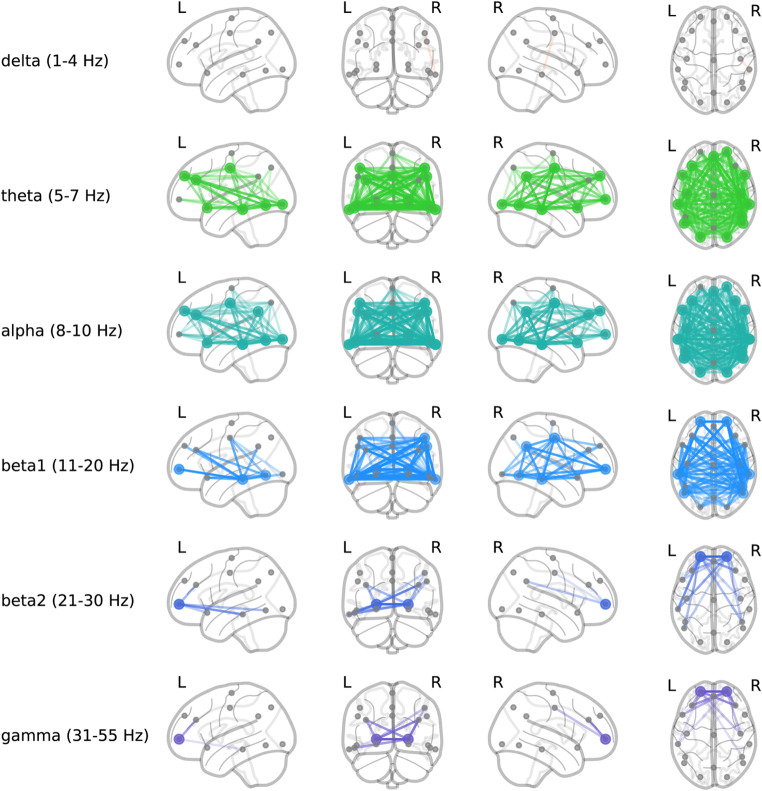
Brain view schematics of learned spectral power and cross-power features associated with NT children (Fig. 5b companion). Associations are averaged over each frequency band and plotted on left sagittal (first column), coronal (second column), right sagittal (third column), and axial (fourth column) views of the brain. Each row represents associations of a different frequency band. Gray dots represent 2D projections of 3D electrode coordinate locations of 10–20 subset electrodes (e.g., gray dots in the axial view indicate Cartesian electrode coordinates). Colored, circular markers plotted over electrode locations denote power associated with NT individuals, and colored lines spanning between electrode locations denote cross-power associated with NT individuals. Features within the top 5% of associations were plotted for clarity.

Model coefficients learned for each repetition of disjoint data splits were averaged over repetitions to visualize associations. To interpret general spectral associations independent of spatial characteristics, coefficient values of features corresponding to a given frequency were summed together. The sum of the associations as a function of frequency is plotted in Fig. 4. Positive-valued coefficients were interpreted as being associated with autism (the positive class in our model) while negative coefficient values were interpreted as associated with NT. In general, lower frequency features (5–10 Hz) were associated with NT, while higher frequency features (> 40 Hz) were associated with autism diagnosis. Chord plots of coefficient associations corresponding to models selected according to model robustness are plotted in Fig. 5. Chord plots reveal that higher frequency features associated with brain regions corresponding to the occipital lobe were highly associated with autism diagnosis (Fig. 5a). Chord plots of coefficient associations corresponding to models selected according to predictive performance are plotted in Fig. 6, which we note have much more scattered frequency and region patterns than Fig. 5. As an alternative method of visualizing associations, brain view schematics of coefficient associations to autism and NT averaged over canonical EEG frequency bands are plotted in Figs. 7 and 8, respectively. EEG bands were defined to the following frequency ranges: delta (1–4 Hz), theta (5–7 Hz), alpha (8–10 Hz), beta1 (11–20 Hz), beta2 (21–30 Hz), and gamma (31–55 Hz). For both chord and brain view schematics plots, the top 5% of coefficient values were plotted for sake of plot clarity.

### Evaluating for study-specific effects

**Figure 9:**
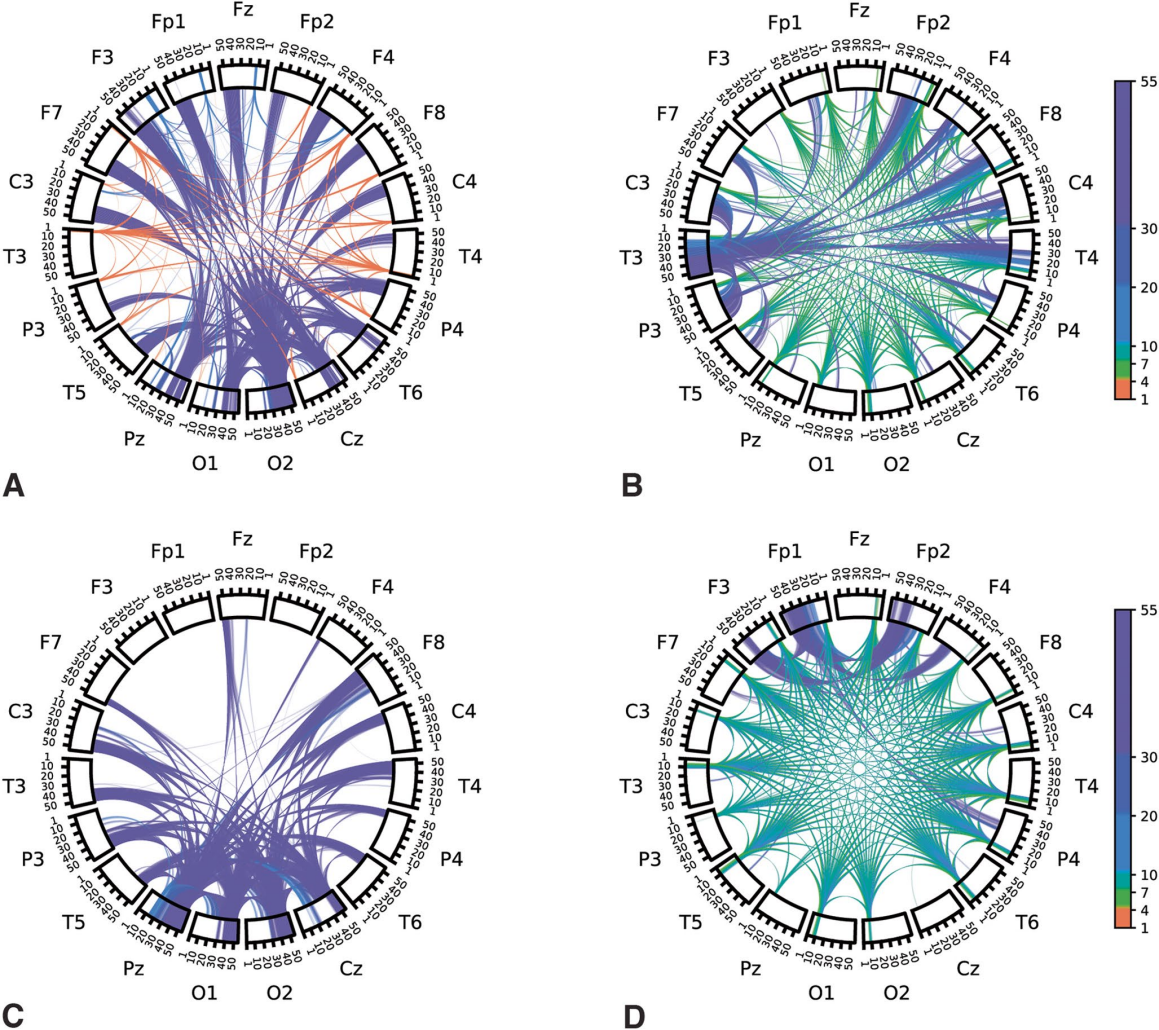
(**a**) Chord plot of learned spectral power and cross-power features associated with children diagnosed with autism in study 1 and (**b**) children diagnosed as NT in study 1. (**c**) Chord plot of learned spectral power and cross-power features associated with children diagnosed with autism in studies 2, 3, and 4 and (**d**) children diagnosed as NT in studies 2, 3, and 4. A threshold was applied in which the top 5% percent of coefficient values were visualized to aid in visual clarity.

We conducted an additional analysis to ensure our learned associations were indeed associated with autism diagnosis and not related to study-specific effects. We applied our model robustness evaluation to data split according to study: study 1 (a dataset with both autism and NT individuals) corresponding to one split and aggregated data from studies 2, 3, and 4 corresponding to the other. When trained on study 1 and tested on aggregated data from studies 2, 3, and 4, our ML pipeline (SIRT preprocessing included) achieves a test set AUROC of 0.775. When trained on aggregated data from studies 2, 3, and 4 and tested on study 1, our ML pipeline achieves a test set AUROC of 0.571. This produced an averaged AUROC of 0.673. The Spearman’s rank correlation coefficient between the two learned representations was 0.318. Chord plots corresponding to associations learned on study 1 and studies 2, 3, and 4 are provided in Fig. 9a, b, c and d, respectively. Both sets of associations emphasized higher frequency gamma features (> 30 Hz) associated with more posterior regions of the brain as features positively associated with autism (Fig. 9a and c). Conversely, both sets of associations identified features in the alpha frequencies (8–10 Hz) as negatively associated with autism (Fig. 9b and d).

### Correlations between EEG features and clinical variables

We next examined whether the EEG features derived from the model correlated with several clinical variables. Correlation analysis revealed that diagnosis prediction logits were significantly correlated with participant age. Full correlation analysis results are reported in Table 3.

**Table 3:** Pearson correlation coefficients and corresponding *p-*values of prediction logit scores and participant age and full scale IQ. A Bonferroni correction was applied to correct for multiple comparisons.

Variable/Assessment Name	Correlation	Raw *p*-value	Corrected *p*-value
Age	1.950 ⋅ 10^−1^	*p* < .001	*p* = .013
FSIQ	-1.034 ⋅ 10^−1^	*p* = .077	*p* = 1

*Note.* Significance was determined according to a threshold level of *α* = 0.05. Abbreviations: Full Scale Intelligence Quotient (FSIQ).

## Discussion

Our results emphasize the importance of model selection procedures when striving for reproducible and interpretable associations between distinctive EEG features and autism, both quantitatively and qualitatively. Selecting models for overall model robustness according to our proposed model selection procedure led to a sizeable increase in the quantitative reproducibility of learned associations, and therefore overall model robustness scores. This is reflected in the visualizations of the learned associations. Associations corresponding to models selected for overall robustness (Fig. 5) were interpretable and exhibit continuity in features of adjacent frequencies. Conversely, visualization of associations corresponding to models selected according to model based on prediction alone (Fig. 6) were qualitatively less interpretable, and therefore more difficult to understand in the context of previous literature. SIRT preprocessing led to a 25% increase in representational reproducibility and a nearly 30% increase in model robustness. We posit that preprocessing via SIRT partially mitigated the domain shift observed in spectral features of neural activity derived from different individuals, thus resulting in improved reproducibility and robustness. This improved robustness can in turn lead to improved confidence in interpretations and subsequent hypotheses and experiments derived from the associations learned here. We note that using robustness for model hyperparameter selection slightly decreases predictive performance, but we believe a model with high robustness (i.e., predictivity *and* association reproducibility) is important for deriving subsequent hypotheses and guiding future experiments, which was the goal of the work here. There are contexts in which a greater emphasis on prediction would be appropriate (e.g., for autism screening).

When we performed model selection based on model robustness, we found that increased RS EEG activity in higher bands of gamma frequencies (> 40 Hz) was strongly associated with autism (Figs. 4, 5a and 7). Differences in gamma activity in autistic individuals have been noted by previous studies^44–47^. found the same general association – *increased* gamma activity – in RS EEG activity of autistic children. Furthermore, our learned associations revealed RS EEG activity in theta and alpha frequencies (5–10 Hz) was found to be strongly associated with NT classification (Figs. 4, 5b and 8). This aligns with the trend noted in the literature of reduced alpha power in autistic children relative to NT children^3,48^ as well as reduced alpha connectivity in autistic children^7,8,49^. Notably, a meta-analysis of 41 studies of autism RS EEG power associations revealed that autistic individuals exhibited reduced relative alpha power and increased gamma power^50^. Such findings from the RS EEG literature, which aligned with our learned associations, have been hypothesized to reflect altered ratios of neural excitation and inhibition^51,52^. Observations of increased ratios of neural excitation/inhibition systems in autistic individuals is hypothesized to arise from atypicalities in GABA-related neurotransmitter systems that bias functional neural activity toward gamma-mediated “excitation” and away from alpha-mediated “inhibition”^3,50,53^. We re-emphasize that the relation of our learned associations is only possible through careful selection of models in a manner that also incorporates quantitative measures of the reproducibility of associations. We emphasize that selecting only for predictive performance did not have obvious patterns consistent with the literature, and the frequencies and regions were highly variable (Fig. 6). Thus, our novel contribution is not the specific neural features which we found were associated with autism, which align well with previous studies, but rather the method by which these associations were derived which allow for greater confidence in their robustness and replicability in future studies. We encourage future studies to utilize similar methods to promote greater reproducibility across autism EEG biomarkers studies.

Our findings also align with studies of the genetic basis of autism. Autism is considered to be highly heritable^54,55^. Studies investigating genetic signatures associated with autism have noted transcriptomic changes that are potentially aligned with the theory of altered excitation/inhibition systems^56^. found widespread differences in RNA expression across the cortex in individuals with autism. Specifically, the study noted “an anterior-to-posterior gradient, with the most pronounced differences in primary visual cortex.” The authors posit that this pattern reflects changes in gene expression specific to excitatory cells (e.g., neurons and glia) in particular. The anterior-to-posterior gradient of increasing transcriptomic differences noted by^56^ aligns with our learned gamma band features associated with autism (Fig. 7, bottom row, fourth column). The putative connection of gamma band oscillations to the mediation of excitatory systems further suggests a potential connection, as the noted transcriptomic differences were hypothesized to be related to excitatory cells. This potential relation of the associations learned here and noted transcriptomic differences may further motivate future studies to explicitly study neural function as it relates to the genetic basis of autism.

For the autistic children in our study, differences in neural activity were evident in occipital and parietal gamma, particularly connectivity between occipital/posterior regions and parietal regions (Figs. 5a and 7). ^57^ found similar patterns of power associations to autism, noting “greater high-frequency activity… that became more pronounced from anterior to posterior and was most evident in parietal regions.” This result partially overlaps with previous findings from^46^ of elevated gamma oscillatory activity; however, their study noted significantly higher gamma power in frontal, parietal, and temporal regions, not occipital. For the NT children in our study, neural activity was characterized by a pattern of highly symmetrized, cross-hemisphere connectivity between temporal regions in the theta and alpha frequencies. The axial brain views (fourth, right-most column) of theta and alpha band activity depicted in Fig. 8 emphasize the intrahemispheric, highly symmetrized connectivity associated with RS neural activity in NT children. A similar result was also found by^2^, who found that intrahemispheric theta coherence was more pronounced in NT controls compared to autistic children.

An intriguing aspect of the learned associations with autism was the emphasis of high frequency power and cross-power in occipital regions. A core feature of autism is hyper- or hyporeactivity to sensory input or unusual interests in sensory aspects of the environment^58,59^. It is possible that our experimental approach elicited differences in how autistic and NT participants attend to and/or process dynamic visual stimuli, thus reflected by features associated with the occipital lobe. Previous studies have suggested that gamma band oscillations reflect cortical activation^60^, with additional work noting the association of gamma activity with aspects of visual attention^61,62^. Specifically, associations learned here could reflect differences in cortical activation in autistic children when attending to dynamic visual stimuli. This is of note as differences in attentive behaviors of autistic children has been described in the literature^63–65^, and identifying the neural basis of these behavioral differences is of great interest to the autism research community.

Lack of significant correlation of prediction logits with participant IQ provided reassurance that our model did not pick up on concomitant variance related to participant intellectual ability which was correlated with diagnosis. Age was found to be positively correlated with the output of our model (e.g., predicted likelihood of autism diagnosis). The strong correlation of prediction logits and age could signify that the learned spectral associations grow stronger/more pronounced as children age and their brains continue to follow their respective developmental trajectories (i.e., a NT developmental trajectory vs. the trajectory associated with autism). This would be in line with findings in the literature, which note distinct neurological developmental trajectories in children with autism, especially in earlier developmental years^66,67^.

The data from studies composed entirely of a single diagnostic group (i.e., study 2 (NT only), study 3 (autism only), and study 4 (autism only)) present a possible limitation concerning the interpretation of our learned associations. It is possible to learn linear associations that detail study-specific differences rather than diagnostic differences. The analysis to evaluate for study-specific effects suggested that domain shifts attributable to study-specific differences may be present in our data, as the predictive performance and representational reproducibility were lower than that achieved when data from all four datasets are present in each disjoint split. However, we noted that the predictive performance achieved in this analysis was comparable to that achieved in the model robustness evaluation reported in Table 2. Additionally, a positive correlation between associations learned with respect to study 1 and studies 2, 3, and 4 indicated the reported associations generalize across studies. This was further confirmed qualitatively, as the chord plots shown in a newly provided Fig. 9 show that both sets of associations share the same general features. Both sets of features positively associated with autism (Fig. 9a and c) included higher frequency gamma features (> 30 Hz) associated with more posterior regions of the brain. Conversely, both sets of features negatively associated with autism (Fig. 9b and d) primarily emphasized features in the alpha frequencies (8–10 Hz). From this analysis, we concluded that study-specific differences were likely present in the data; however, the general associations reported in this work were preserved across datasets, indicating that we have identified signal associated with clinical diagnosis of autism.

A further limitation of this study is that learned associations likely better characterize the neural differences in autistic boys due to the approximately 3-to-1 ratio of males to females in this study. Although the ratio of males to females in this study is imbalanced, we note that, at the time of this writing, autism has a prevalence ratio of 3 males for every 1 female diagnosed^68^ so the sample considered here is representative of the relative prevalence of autism in males vs. females. Additionally, cross-power does not capture directional flow of information. Directed measures of functional connectivity (e.g., phase slope index^69^) could provide a more detailed characterization of autistic brain function. Cross-power also does not characterize possible cross-frequency interactions. Atypicalities in measures that examines cross-frequency interactions, such as phase-amplitude coupling, have been noted in RS neural activity in children with autism^70,71^. Thus, incorporating features that characterize cross-frequency interactions into future modeling could provide better discrimination in prediction of autism diagnosis and help further characterize neural etiology. Another limitation stems from the nature of the EEG recording modality. EEG primarily picks up population-level neuronal activity from the outermost layers of the cortical brain. Furthermore, EEG details macroscale, population-level neural activity and does provide information on more detailed neural activity such as spike patterns from individual neurons.

We examined RS neural activity collected from a multi-study pediatric cohort composed of autistic and NT children. We identified a distinct and interpretable profile of spectral power and cross-power associated with RS neural activity of children with autism. The learned profile detailed both spatial and spectral neural signal features associated with autism and aligned well with findings from the literature, namely reduced alpha band power and connectivity in autistic individuals and elevated gamma band activity. Notably, the interpretability of our learned associations and the alignment with results from previous literature was only possible when selecting models based on both predictive performance *and* reproducibility of associations, as we proposed. Additionally, we demonstrated that our ML approach resulted in modest improvements in identifying reproducible associations. While our modeling pipeline demonstrated partial diagnostic discriminative ability, we noted several limitations that precluded a comprehensive characterization of neural activity in autistic children. Thus, further research is warranted to find more diagnostically discriminative and generalizable neural biomarkers of autism, and to continue pursuit of robust characterization of the underlying neural mechanisms of autism.

## Supplementary Information

Supplementary Material 1.

Supplementary Material 2.

## Data Availability

Data are available through collaboration with authors. To request access to data or inquire about possible collaboration, please contact the corresponding author Dr. David Carlson (david.carlson [at] duke.edu).
