# Supplementary material for: Model selection to achieve reproducible associations between resting state EEG features and autism
Source: Sci Rep. 2024 Oct 25;14:25301. doi: 10.1038/s41598-024-76659-5 (PMC11511871; doi:10.1038/s41598-024-76659-5)
Supplement: Supplementary file 2 — Supplementary Material 2. [file 41598_2024_76659_MOESM2_ESM.pdf]

Supplemental Video: Brain view schematics animations of learned spectral power and cross-power features associated with NT children (top row) and children diagnosed with autism (bottom row). Associations are animated with respect to increasing frequency and plotted on left sagittal (first column), coronal (second column), right sagittal (third column), and axial (fourth column) views of the brain. Gray dots represent 2D projections of 3D electrode coordinate locations of 10-20 subset electrodes (e.g., gray dots in the axial view indicate Cartesian electrode coordinates). Colored, circular markers plotted over electrode locations denote power associated with diagnosis, and colored lines spanning between electrode locations denote cross-power associated with diagnosis. Features within the top 5% of associations (for both NT and autism) are plotted for clarity.
